# Effects of a postural cueing for head and neck posture on lumbar lordosis angles in healthy young and older adults: a preliminary study

**DOI:** 10.1186/s13018-022-03090-9

**Published:** 2022-04-04

**Authors:** Meiling Zhai, Yongchao Huang, Shi Zhou, Jiayun Feng, Chaolei Pei, Li Wen

**Affiliations:** 1 School of Sports and Health, Nanjing Sport Institute, No.8 Linggusi road, Nanjing, 210014 Jiangsu China; 2 Institute of Exercise and Health, Tianjin University of Sport, No.16 Donghai Road, West Tuanbo New Town, Jinghai District, Tianjin, 301617 China; 3 Sport and Exercise Science, Faculty of Health, Southern Cross University, P Block, Military Road, East Lismore, NSW 2480 Australia; 4 Institute of Sports Training, Tianjin University of Sport, No.16 Donghai Road, West Tuanbo New Town, Jinghai District, Tianjin, 301617 China; 5 Institute of Sports Training, Shanxi Normal University, No.339 Taiyu Road, Xiaodian District, Taiyuan City, 030000 China

**Keywords:** Posture, Cervical anterior angle, Lumbar lordosis angle, Chin tuck

## Abstract

**Background:**

Postural rehabilitation plays an important role in the treatment of non-specific low back pain. Although pelvic inclination has been widely used to improve lumbar lordosis, the effect of cervical anterior inclination on lumbar lordosis in young and older adults in sitting and standing posture is still unclear. This preliminary study was designed to examine the influence of changing the cervical anterior angle on the lumbar lordosis angle, through alterations of the head position under the natural sitting and standing conditions, aiming to provide a basis for establishing a new postural rehabilitation strategy.

**Methods:**

Thirty-six young (24.0 ± 2.2 years, 14 females and 22 males) and 38 older (68.4 ± 5.9 years, 36 females and 2 males) healthy adults participated in this study. The four spinal regional angles—cervical anterior angle, thoracic kyphosis angle, lumbar lordosis angle, and pelvic forward inclination angle, were measured in standing and relaxed sitting postures to determine the effects of a postural cueing for the head and neck posture, “inclining head backward and performing chin tuck,” on lumbar lordosis angle.

**Results:**

In the standing posture, the pelvic forward inclination angle in the older adult group was significantly smaller (*P* < 0.001, by ANOVA) than that in the young adult group and increased significantly (*P* < 0.001) in response to the postural cueing. In addition, the thoracic kyphosis angle in the standing (*P* = 0.001) and sitting (*P* = 0.003) positions was significantly reduced in response to the postural cueing. However, the lumbar lordosis angle in response to the postural cueing increased significantly in both the standing position (*P* < 0.001) and sitting position (*P* < 0.001).

**Conclusion:**

The results suggest that increasing the cervical anterior angle can increase the lumbar lordosis angle, and the cervical anterior inclination can be used as an alternative to pelvic forward inclination to improve the lumbar lordosis angle. Furthermore, the change in head and neck posture can reduce the thoracic kyphosis angle, making it possible to establish a new noninvasive body posture rehabilitation strategy.

**Supplementary Information:**

The online version contains supplementary material available at 10.1186/s13018-022-03090-9.

## Background

Manipulation of lumbar lordosis (LL) has a clinical significance because it is the basis for the treatment and prevention of low back pain (LBP) [1]. According to epidemiological studies, 50% to 80% of people experience an LBP at least once in their lifetime [2]. In addition, the prevalence of LBP increases with age [3].

The reduction of lumbar lordosis angle (LLA) is considered to be one of the causes of LBP [4]. Pries et al. conducted a study on symptomatic patients aged 20–75 years and showed that the older adults commonly had the phenomenon of stooping due to physical degeneration or iatrogenic factors, and the LLA of the older adults was significantly smaller than that of the young adults during standing [5]. In addition, sitting posture is also a potential cause of LBP [6]. When sitting, the pelvis rotates backward and the LL becomes flat. This posture increases the pressure on the passive components in the back of the spine, which in turn may cause LBP [7]. A study on 459 university students with LBP showed that the prevalence rate of LBP among the participants was 75.8%, and the prevalence rate of chronic LBP was 12.4% [8]. For the elderly, due to the decline in physical function, half of their waking time is in a sitting position [9], so the sitting position in daily life is a vital contributor to LL status [10].

Therefore, when there is an abnormality in LL, how to correct it has become a practical question in research. Previous studies have shown that the pelvic forward inclination can extend the lumbar spine and increase the LLA [11]. However, Claeys et al. showed that posture correction could not be initiated by optimizing the position of the pelvis when standing [12]. In addition, individuals suffering from LBP also have an increased risk of persistent neck pain [13], and the pain is associated with weakened strength and reduced activation in the muscles acting on the shoulder and neck [14]. However, to our knowledge, little research has been reported on improving the LLA and preventing and treating lumbar injury through the relationship between the neck and lumbar.

According to the above background, the purpose of this research was to explore the effect of a postural cueing "inclining head backward and performing chin tuck" on LL with the objective of reducing cervical anterior inclination. It was hypothesized that the LLA could be improved by manipulating the frontal inclination of the cervical vertebrae. The results of the research would set a basis for improving the stooping hunchback of the older adults and young people's desk sitting posture, preventing and treating lower back pain, and establishing a new body posture rehabilitation strategy.

## Methods

This study used a self-control design for a within-group comparison of the spine angle changes pre and post the intervention in healthy adults, as well as a cross-sectional design for a between-group comparison for the effect of age, to verify the effectiveness of postural cueing in improving LLA.

### Participants

A total of 74 healthy participants (36 young adults, including 14 females and 22 males; 38 older adults, including 36 females and 2 males) volunteered to participate in this study. The young adults had an average age of 24.0 ± 2.2 years, weight of 67.2 ± 11.8 kg, height of 1.72 ± 7.9 m, and body mass index of 22.4 ± 2.9 kg/m^2^, while the older adults had an average age of 68.4 ± 5.9 years, weight of 61.6 ± 8.4 kg, height of 1.6 ± 4.9 m, and body mass index of 24.1 ± 3.0 kg/m^2^.

The participants were recruited from the local community, with the young adults who were university students and the older adults were local residents of Tianjin City. We use the short musculoskeletal functional assessments (SMFA) [15] to screen participants’ musculoskeletal function for potential risk factors. The results showed that all participants had no severe spinal and lower limb musculoskeletal problems during the past two months that would prevented them from participation in this study. The experimental procedures were approved by the Ethics Committee of Tianjin University of Sport (approval number TJUS2019032), and all participants gave their written informed consent prior to the commencement of the study.

### Spine angle assessments

**Fig. 1:**
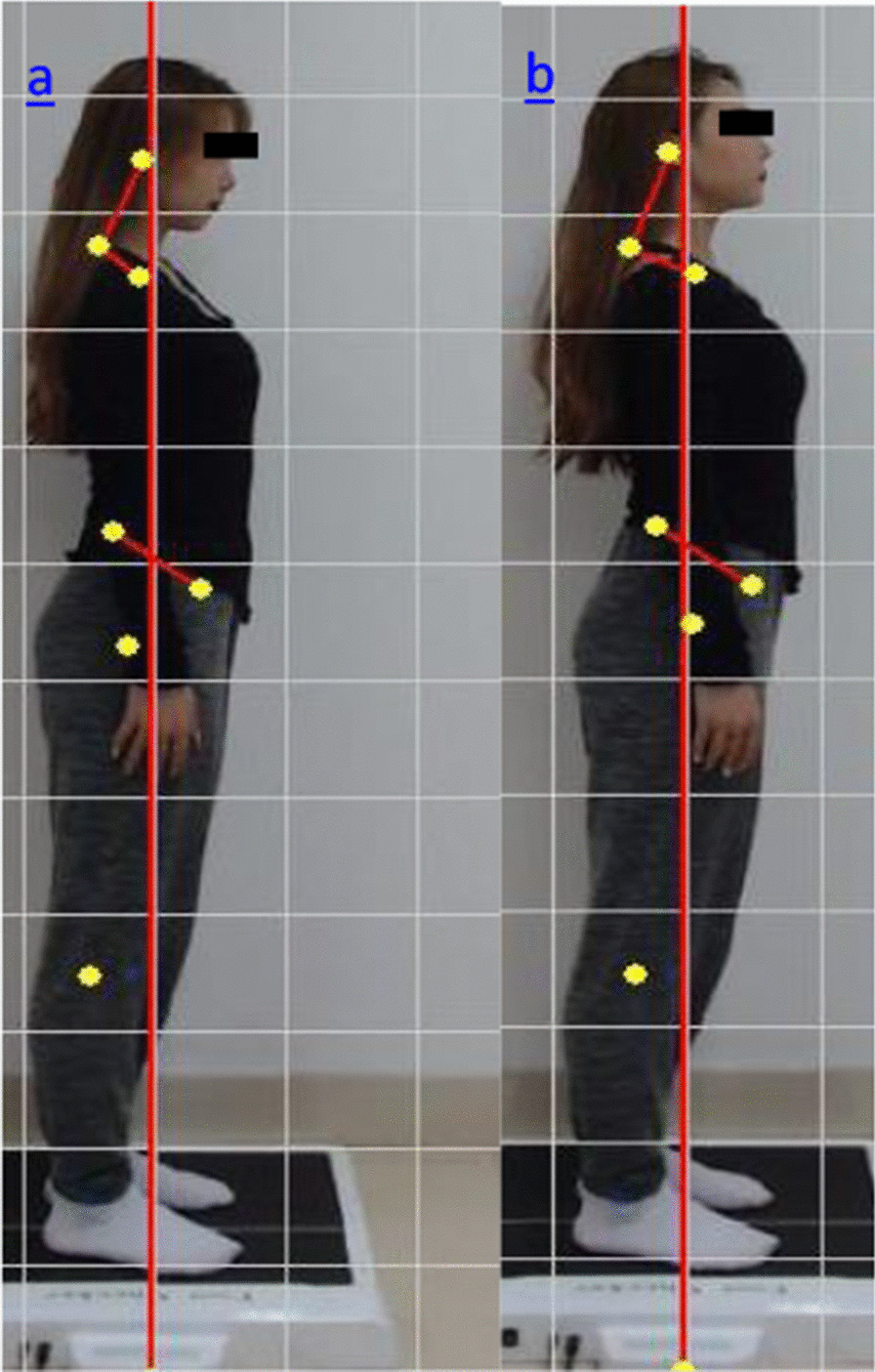
**a** Posture in natural standing. **b** Posture after the postural cueing

A PA200 posture evaluation system (BigSports, Japan) was used to measure the cervical anterior angle (CAA) and pelvic foreword inclination angle (PFIA). A digital camera (Sony A200 DSLR-A200K Scope DT18-70 mm F3.5–5.6; Tokyo, Japan) was placed on a tripod, 2.41 m away from the participant as per PA200 specification of equipment installation requirements, to take photographs of the posture in natural standing and the posture after the postural cueing. Figure 1 shows the standing posture pre and post the verbal cueing.

Participants wore tight clothing. The markers for photoimage analysis were affixed over the bony landmarks on the cloth and skin, including ear foramen, spinous process of the cervical vertebra C7, greater tubercle of the humerus, anterior superior iliac spine (ASIS), and posterior superior iliac spine (PSIS), as utilized in previous studies [16, 17].

For the natural standing position, the participants were asked to take their normal standing position, and standing on the plantar pressure plate, with their feet 10 cm apart, and arms relaxed along the side of the body, with their gaze horizontally on the camera in front.

For the natural sitting position, the participants (young adult group only) were asked to sit naturally in the chair, with their arms naturally placed on the table, and their gaze horizontally on the camera in front. Using external markers in combination with digital photographs has been proved to be reliable and valid in postural evaluation [18–20] and has been utilized in our previous studies [21]. The method of taking photographs for spine angle analysis, as a part of the noninvasive whole-body posture assessment, can help avoid radiographic exposure [22]. Being aware that all methods may have some pros and cons, it was the choice of this study considering its low cost, noninvasive nature and acceptable reliability [23].

**Fig. 2:**
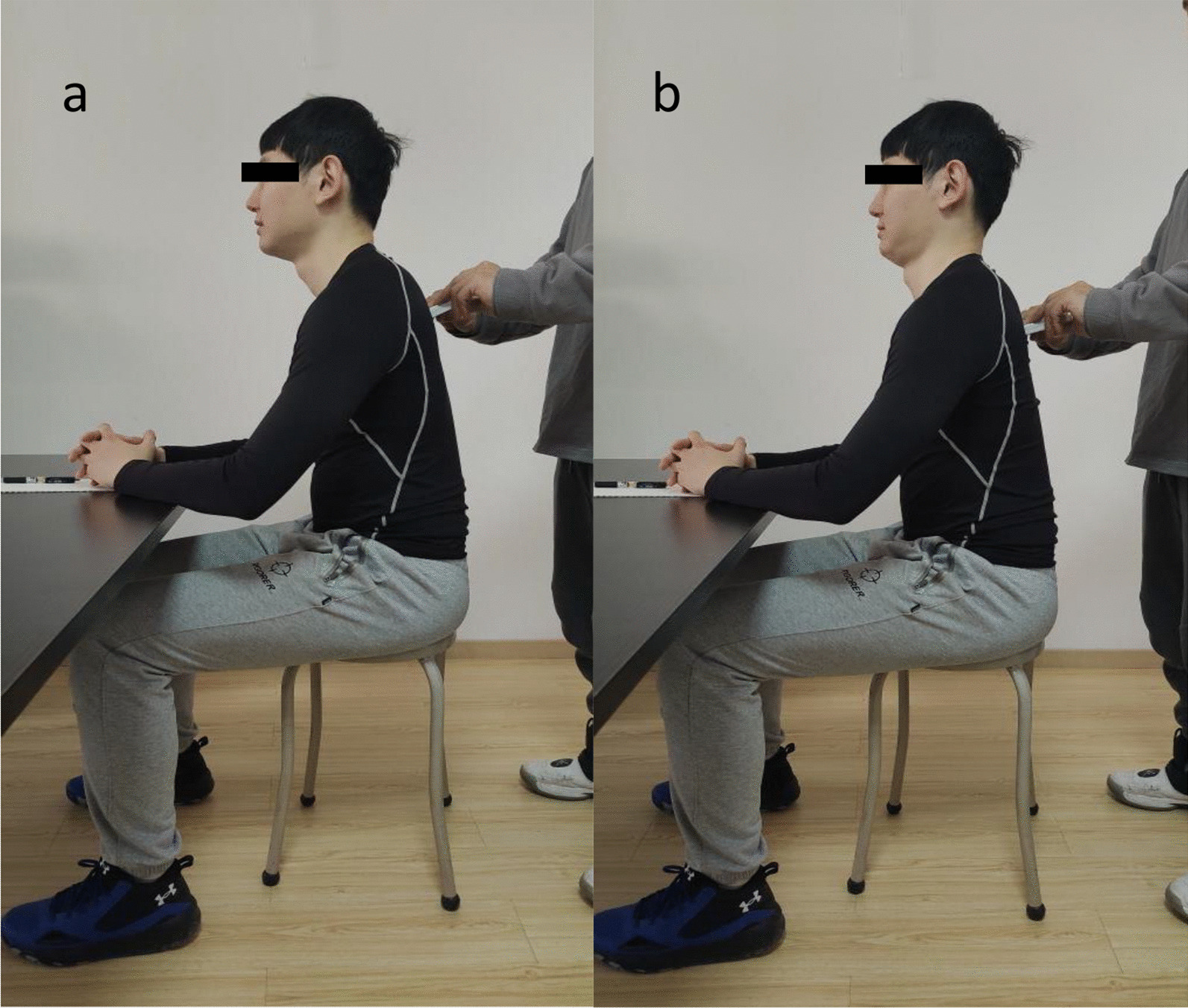
**a** Measure the participants' spine angle pre the intervention. **b** Measure the participants' spine angle post the intervention

The SpineScan spine tester (Sunlight, Israel) was used to measure the participants' spine angle pre and post the postural cueing intervention (Fig. 2). When measuring, the electronic spine measuring instrument was slid along the spine from above C1 to S1, and the thoracic kyphosis angle (TKA) and LLA in the natural standing and sitting position, and the CAA in the natural sitting position were measured. Studies have proved that the SpineScan is reliable in measuring spine angle under the operation of the same user [24].

### The postural cueing

Standing posture was assessed by photographing (Fig. 1), and sitting posture was assessed by physical measurement (Fig. 2), pre and post the postural cueing. The participants adjusted their posture according to the verbal instruction of " inclining head backward and performing chin tuck," which aimed to increase the CAA.

### Muscle tension simulation

Muscle tension was evaluated by computer simulation. The PA200 posture evaluation system included an established software for assessing postural changes caused by muscle imbalance (Additional file 1). According to the body posture measured in this study, the muscle tension changes were simulated by using the software and presented in the images of the participants (Fig. 4).

**Fig. 3:**
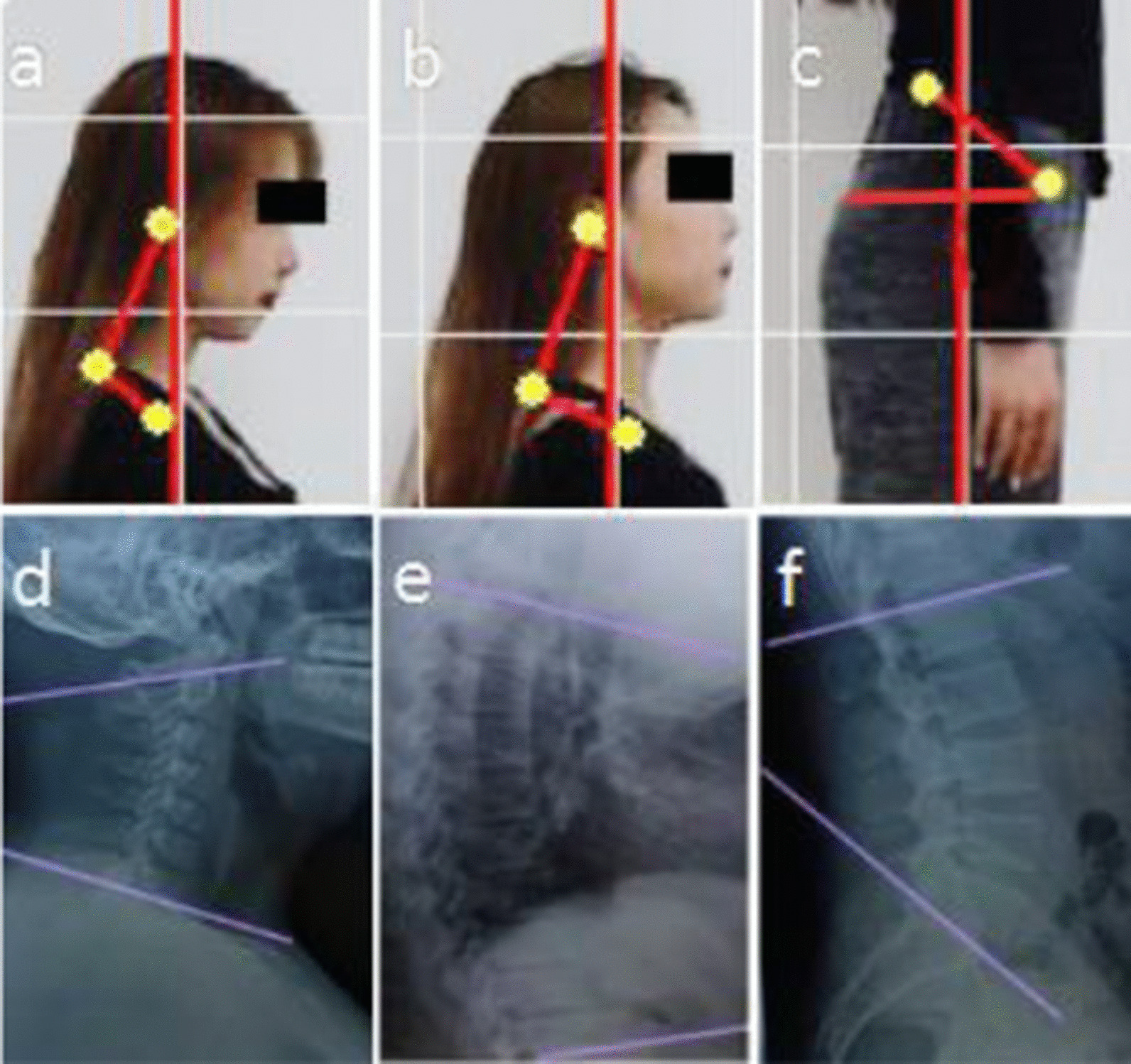
**a** Standing CAA. The definition of CAA is the angle between two lines with Tragus of Ear and Spinous process of C7 and Spinous process of C7 and greater tuberosity of humerus. **b** Raising CAA. The definition of raising CAA is the same as the standing CAA. **c** PFIA. Definition of PFIA is the ASIS-PSIS-horizontal plane. **d** Sitting CAA. Definition of Sitting CAA is the angle between lower endplates of C1 and that of C7. **e** TKA. Definition of TKA is the angle between T4 superior endplates and T12 lower endplates. **f** LLA. Definition of LLA is the angle between superior endplates of L1 and that of S1

**Fig. 4:**
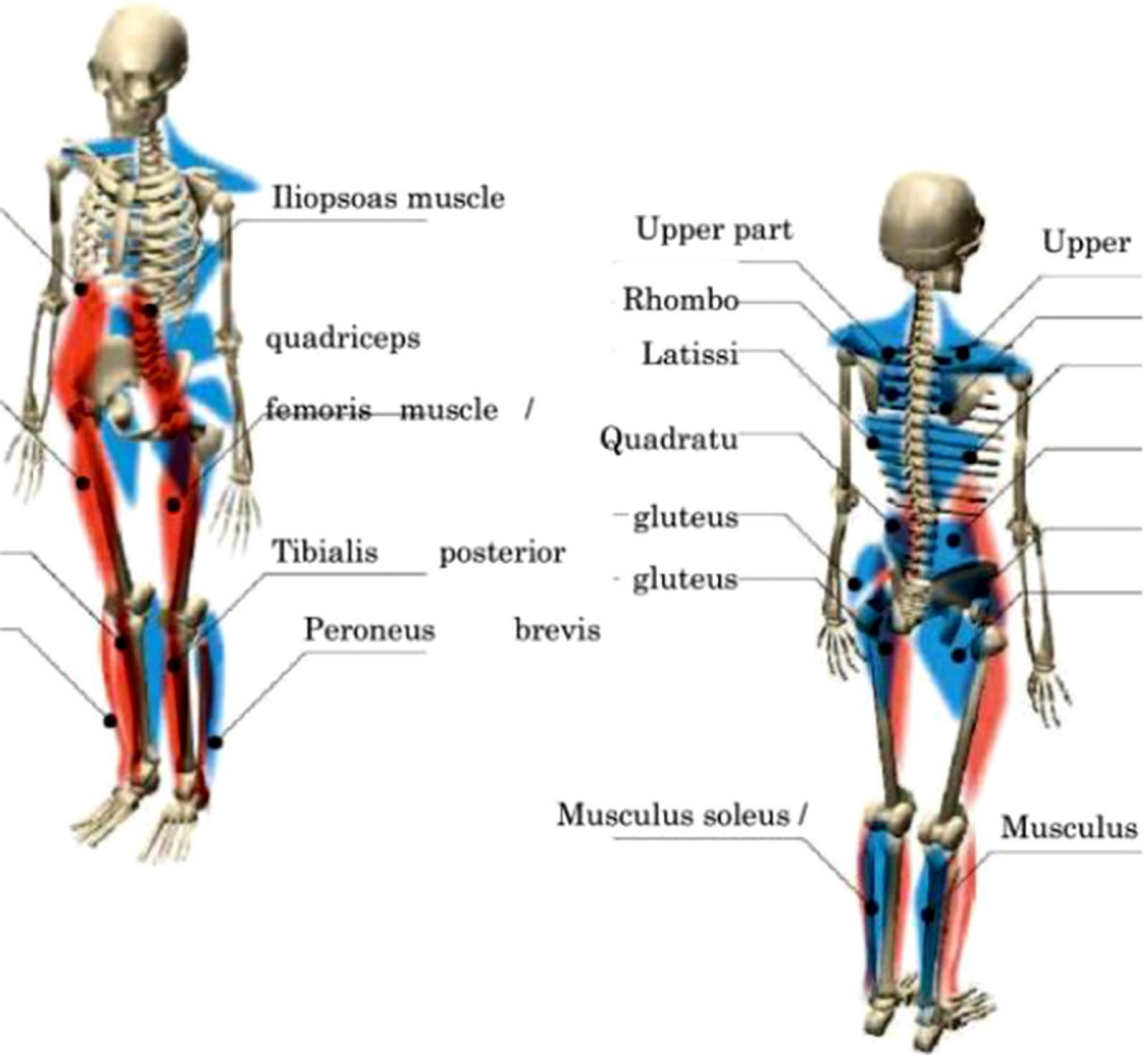
Muscle tension simulation image (generated by PA200)

Figure 3 shows the definition of all posture angles. Figure 4 is an illustration of the muscle tension estimation, in which the muscle tension levels were represented by colors, with the red color represented excessive lengthening of the muscle, blue color indicated excessive shortening of the muscle, and the muscle area without color indicates the normal muscle state.

### Statistical analysis

The data were analyzed by IBM SPSS Statistics software (ver.23.0, New York, USA). Each variable was described as the mean ± SD. Data distribution normality was checked by applying the Shapiro–Wilk test, and the equal variance was judged by the Levene’s variance homogeneity test. To test the validity of the postural cueing, CAA measured pre and post the cueing intervention was analyzed by paired t tests. The mixed-model analysis of variance (ANOVA) was used for between-groups (age) and within-group (intervention) comparisons. The Wilcoxon signed-rank test was used to compare the spinal angle pre- and post-intervention under sitting conditions in the young adult group. At the same time, Pearson correlation analysis was carried out on demographic factors such as gender and age in relation to the measured variables. To test the effect of the intervention on muscle tension, ordered logistic regression analysis was used. Statistical significance was set at *P* < 0.05.

All participants’ postural assessments were performed by one experienced researcher. The researcher demonstrated a good intra-measurer correlation coefficient (ICC) ranged from 0.75 to 0.90. The ICC is similar to that reported in the literature using the same measurement methods [25].

The sample size required for adequate statistical power was estimated using G*Power ver.3.1.9.2, under the assumptions of α level 0.05, power 0.8, and effect size 0.25. The minimum sample size required was estimated as 34 for two-way ANOVA.

## Results

### Analysis of the difference of spine angle under natural standing and sitting posture between the young and older adult groups

It can be seen from Table 1 that the CAAs of the three assessments (young and older standing, and young sitting) post the postural cueing were all greater than that pre the intervention, and the difference was statistically significant (*t* = 5.924, *P* < 0.001; *t* = 6.291, *P* < 0.001; *t* = 5.924, *P* < 0.001, respectively), indicating that the postural cueing had a significant effect on the posture.

**Table 1:** Comparisons of CAA pre and post the postural cueing

Group	CAAs (degree)				
	Pre-intervention	Post-intervention	Post–pre difference	*t*	*P *value
Standing young (*n* = 36)	90.67 ± 9.67	97.22 ± 9.34	6.56 ± 6.64	5.924	< 0.001
Standing older (*n* = 38)	91.34 ± 9.92	99.05 ± 12.81	7.71 ± 7.55	6.291	< 0.001
Seated young (*n* = 36)	8.42 ± 3.26	15.14 ± 4.64	6.72 ± 3.94	10.238	< 0.001

Table 2 shows that the age and intervention had no statistically significant interaction in the measurement of spine angle (*F* = 0.241, *P* = 0.625; *F* = 0.029, *P* = 0.865; *F* = 1.091, *P* = 0.300). The comparison between groups showed that the TKA of the elderly was larger than that of the young (*F* = 3.265, *P* = 0.075), the LLA was smaller than that of the young (*F* = 2.793, *P* = 0.099), but these differences were not statistically significant, while the anterior inclination angle of the pelvis was significantly larger in the older adults than that of the young group (*F* = 187.098, *P* < 0.001). The comparison for pre and post the intervention showed that the TKA was significantly reduced (*F* = 12.522, *P* = 0.001); the LLA was significantly increased (*F* = 66.571, *P* < 0.001); and the PFIA significantly increased (*F* = 72.466, *P* < 0.001).

**Table 2:** Comparisons of thoracic, lumbar, and pelvic angles between the age groups and pre and post the intervention

Intervention	TKA (degree)		LLA (degree)		PFIA (degree)	
	Young group (*n* = 36)	Oder group (*n* = 38)	Young group *(n* = 36)	Oder group (*n* = 38)	Young group (*n* = 36)	Oder group (*n* = 38)
Pre-intervention	23.28 ± 4.11	25.68 ± 6.02	9.85 ± 5.73	7.96 ± 3.14	22.97 ± 5.01	9.8 ± 3.22
Post-intervention	21.33 ± 6.31	23.09 ± 5.91	13.31 ± 6.52	11.28 ± 5.48	25.40 ± 4.54	12.91 ± 4.17
Comparison between groups (*F*, *P*)	3.265, 0.075		2.793, 0.099		187.098, 0.000	
Comparison pre- and post-intervention (*F*, *P*)	12.522, 0.001		66.571, 0.000		72.466, 0.000	
Interaction of age and intervention (*F*, *P*)	0.241, 0.625		0.029, 0.865		1.091, 0.300	

Table 3 shows the kyphosis angle of thoracic vertebrae in sitting position decreased significantly post-postural cueing (*Z* = − 2.957, *P* = 0.003). The postural cueing resulted in an increase in LLA in sitting position (*Z* = -5.026, *P* < 0.001).

**Table 3:** Comparisons of TK and LL pre- and post-sitting posture intervention

Spine angle	Pre-intervention	Post-intervention	intervention (post–pre)	*Z*	Significant (double tail)
TKA in sitting position	23.460	22.890	− 0.795	− 2.957^a^	0.003
LLA in sitting position	5.540	8.130	2.380	− 5.013^b^	0.000

^a^Based on the positive rank

^b^Based on the negative rank

The correlation coefficients between the spine angle and anthropometric variables, such as age, height, weight, and BMI in standing and sitting positions, are shown in Table 4.It can be seen in Table 4 that CAA was negatively correlated with height (*r* = − 0.351, *P* = 0.002), weight (*r* = − 0.481, *P* < 0.001), and BMI (*r* = − 0.336, *P* = 0.003); TKA was positively correlated with age (*r* = 0.246, *P* = 0.035); PFIA was positively correlated with height (*r* = 0.471, *P* < 0.001), and negatively correlated with age (*r* = − 0.848, *P* < 0.001) and BMI (*r* = − 0.257, *P* = 0.027) in standing posture. But age, height, weight, BMI, and spine angle are not correlated in sitting position (*P* > 0.05).

**Table 4:** Correlation coefficients of spine angle and anthropometrical measurements of the participants in standing and sitting postures

Posture	Spine angle	Age	Height	Weight	BMI
Standing position	CAA	0.012	− .351**	− .481**	− .336**
	TKA	.246*	− 0.179	0.019	0.219
	LLA	− 0.195	− 0.054	− 0.082	− 0.055
	PFIA	− .848**	.471**	0.126	− .257*
Sitting position	CAA	0.109	− 0.190	− 0.040	0.133
	TKA	0.190	− 0.146	− 0.235	− 0.243
	LLA	0.150	− 0.077	− 0.108	− 0.097

*Representative *P* < 0.05

**Representative *P* < 0.01

### Comparison between the older and the young groups

**Fig. 5:**
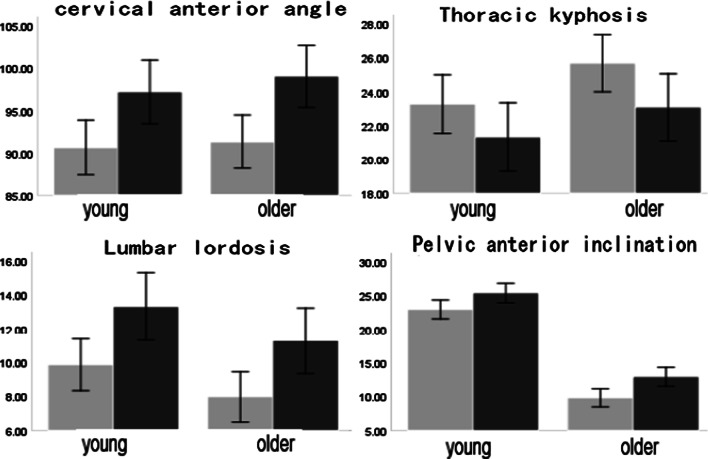
Comparison of the spinal angles between the young and older adults in a standing position (unit: degree) (Gray represents pre the postural cueing and black represents post the postural cueing)

Figure 5 shows that there is no significant difference in CAA between the older and the young adults' pre or post the intervention when standing naturally. The adjustment of the CAA to the spine angle is mainly reflected in the lumbar spine. Although the lordosis angle of the old lumbar spine was smaller than that of the young, the LLA of the older adult group increased significantly post the intervention. In addition, the TKA was also affected by the intervention on the cervical spine. Although the TKA of the older group was larger than that of the young, it was significantly reduced post the intervention. However, although the anterior pelvic angle of the elderly increased significantly post the intervention, the gap was still large compared with that of the young.

Sequential logistic regression analysis was used to analyze the effect of the postural cueing on muscle tension. The results are shown in Table 5. Post the intervention, the OR values of "passive shortening" of the left and right rhomboid muscles were 0.161 times (*X*^*2*^ = 5.072, *P* = 0.024) and 5.939 times (*X*^*2*^ = 4.785, *P* = 0.029) pre the intervention, respectively. The OR values of "passive shortening" of left and right soleus were 6.538 times pre the intervention (*X*^*2*^ = 5.279, *P* = 0.022) and 6.538 times (*X*^*2*^ = 5.279, *P* = 0.022). The OR value of "passive shortening" of the left quadriceps post-intervention was 8.83 times greater than that in pre the intervention (*X*^*2*^ = 7.44, *P* = 0.006), and for the right quadriceps, it was 3.626 times greater than that pre-intervention (*X*^*2*^ = 4.128, *P* = 0.042). However, in the older group only the left trapezius showed a significant change post the intervention, with the OR value of "passive shortening" post-intervention was 0.272 times greater than that of pre-intervention (*X*^*2*^ = 5.64, *P* = 0.018).

**Table 5:** Effects of postural cueing on muscle tension in young and older people

	Muscle	*B*	Wader Chi-square	Significance	Exp(*B*)	95% CI of Exp(*B*)	
						Lower limit	Upper limit
Young	Right trapezius	− 1.079	2.806	0.094	0.340	0.096	1.201
	Left rhomboid muscle	− 1.826	5.072	0.024	0.161	0.033	0.789
	Right rhomboid muscle	1.782	4.785	0.029	5.939	1.204	29.311
	Left latissimus dorsi	0.261	0.317	0.573	1.298	0.524	3.212
	Right latissimus dorsi	− 0.261	0.317	0.573	0.771	0.311	1.907
	Left quadratus lumborum	0.526	1.191	0.275	1.693	0.658	4.355
	Right quadratus lumbar muscle	− 0.985	3.779	0.052	0.374	0.138	1.008
	Left soleus muscle	1.878	5.279	0.022	6.538	1.318	32.442
	Right soleus muscle	1.878	5.279	0.022	6.538	1.318	32.442
	Left iliopsoas	1.124	3.010	0.083	3.077	0.864	10.954
	Right iliopsoas muscle	− 1.124	3.010	0.083	0.325	0.091	1.157
	Left quadriceps	2.178	7.440	0.006	8.83	1.846	42.233
	Right quadriceps	1.288	4.128	0.042	3.626	1.047	12.562
Older	Right trapezius	− 0.575	1.337	0.248	0.563	0.212	1.491
	Left rhomboid muscle	− 1.303	5.640	0.018	0.272	0.093	0.796
	Right rhomboid muscle	0.864	2.883	0.090	2.372	0.875	6.431
	Left latissimus dorsi	− 0.217	0.205	0.651	0.805	0.315	2.059
	Right latissimus dorsi	0.217	0.205	0.651	1.242	0.486	3.177
	Left quadratus lumborum	− 0.318	0.501	0.479	0.727	0.301	1.756
	Right quadratus lumbar muscle	− 0.468	1.096	0.295	0.627	0.261	1.503
	Right hamstring	− 0.292	0.290	0.590	0.747	0.258	2.161
	Left iliopsoas	0.256	0.255	0.613	1.292	0.478	3.494
	Left quadriceps	− 0.660	2.359	0.125	0.517	0.223	1.200
	Right quadriceps	0.460	1.043	0.307	1.584	0.655	3.832

### Comparison between sitting position and standing position

**Fig. 6:**
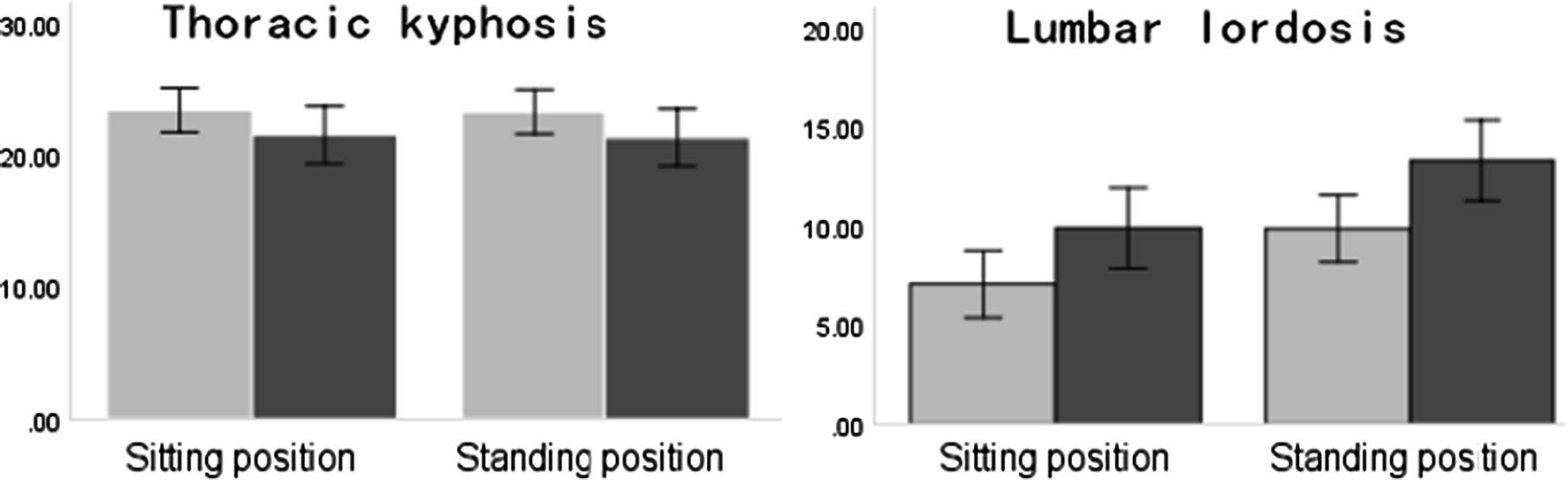
Comparison of sitting and standing position, TKA and LLA in young adults (unit: degree) (Gray represents pre the postural cueing and black represents post the postural cueing)

As seen from Fig. 6, the kyphosis angle of thoracic vertebrae in both standing and sitting positions decreased post the intervention, but there was no significant difference between the two positions. In addition, the LLA in the sitting position was significantly smaller than that in the standing position, and the LLA increased significantly post the intervention.

## Discussion

The main findings of this study include that there was no statistical difference between the thoracic kyphosis and the lumbar lordosis in the standing position, while the pelvic forward inclination of the older group was significantly smaller than that of the young group. Thoracic kyphosis, lumbar lordosis, and pelvic forward inclination were significantly different pre and post the verbal intervention. In addition, in sitting position, the thoracic kyphosis angle and lumbar lordosis angle of the young group were statistically different pre vs post the intervention.

The estimated muscle tension in young and older groups indicated that the verbal intervention did not result in significant changes in muscle tension of the left trapezius muscle, left hamstring muscle, left and right soleus muscle, tibialis anterior muscle, and fibula brevis muscle of both groups. In the young adult group, the left and right rhomboid muscles were significantly elongated after the intervention compared with before intervention whist the left and right soleus muscles and the left and right quadriceps were significantly shortened. However, in the older adult group only the left rhomboid muscle was significantly elongated after the intervention compared with that before the intervention.

### Cervical anterior inclination angle

The increased anterior inclination of the cervical spine would indicate the effectiveness of the verbal intervention in both standing and sitting positions. We compared our results with the reference value of cervical anteversion from other studies. The results showed that the cervical anterior inclination (90.67 ± 9.67 degrees) of the young people in our study was similar to that reported by Zhu et al. [26], while the cervical anterior inclination (91.34 ± 9.92 degrees) of the older people in our study was in contrast to that of Zhou [27]. In addition, the anterior inclination of the cervical spine during sitting in the young adult group was same as that of Zhou et al. [27], which was different from that reported by Hey [28]. The discrepancies might be caused by the research methods employed, because there were obvious differences in the procedures and angle calculation methods of different photogrammetry methods [28], and even if the data collection methods were similar, the calculation methods could be very different [29].

Previous studies have shown that aging is associated with an increase in cervical anteversion relative to the horizontal direction [30]. Our study showed that the mean value of cervical anteversion was larger in the elderly than in the young, but there was no statistically significant difference between the two age groups. However, Kado et al. found that the cervical kyphosis decreased significantly in both gender and age groups under 70 years old, and a significant decrease in cervical lordosis curvature was observed in female group and young group [31]. In this study, due to the uneven gender distribution of the participants, the difference between genders was not assessed.

### Kyphosis angle of thoracic vertebrae

The most obvious and recognizable postural change in the older adult group was an increase in thoracic kyphosis. Our results showed that the 25.42 ± 5.80 degree of thoracic kyphosis in the standing position in the older group was higher than that in the young group (23.28 ± 4.11), although no statistically significance. Hu et al. reported research results showed that the thoracic curvature of the elderly was 27.5 ± 8.5 degrees and the young group was 23.8 ± 8.5 [32], which were similar to our reference value. Furthermore, research by Kuo and colleagues also showed that there was no statistically significant difference between the age groups in standing positions [33]. The reason for this phenomenon may be that the older adults who had a significant increase in thoracic kyphosis might have other health conditions [13] and were excluded from the participation of the study. Study has shown that there is a moderate positive correlation between age and thoracic kyphosis [34], which is consistent with the results of our correlation analysis.

Thoracic kyphosis was significantly reduced post the intervention in both young and older groups. Although the increase in thoracic kyphosis angle is related to aging, it can be changed through the adjustment of body posture. However, there was no statistical difference in thoracic kyphosis angle between sitting position and standing position found in the present study. Different to our findings, Nishida reported that the changes from standing to sitting posture flattened the kyphosis of the thoracic vertebrae [35], which might be due to different standards utilized in postural assessments or internal differences among the participants. Interestingly, we found that the thoracic kyphosis angle in the sitting position was zero. This may be due to that, under the sitting position, the position of the pelvis on the supporting base was relatively fixed [36]. Without the influence from anteversion of the pelvis, the thoracic vertebrae were straightened for compensation. Long-term fixed posture could lead to an increase in back muscle tension and some related disorders such as back pain [37].

### Lumbar lordosis angle

A study reported that aging was related to the decrease or loss of lumbar curvature [38]. Another study reported that the lumbar lordosis angle of the elderly was significantly smaller than that of the young, and the lumbar lordosis angle of the females was larger than that of the males [39]. The effect of BMI on lumbar lordosis angle was reportedly not significant [40]. However, some researchers found that the reduction of these angles mainly occurred in the middle part of the kyphosis, less in the lumbosacral and thoracolumbar transition, and gender only affected the maximum range of upper body extension [41]. Therefore, the locations of the markers and the definition of the angle can also affect the measurement outcomes. If the markers were defined and placed following the same protocol, the results of the current study could be used as a reference for body position evaluation in future studies [42].

Although there appeared to be some differences between the age groups (7.96 ± 3.14 degrees in the elderly, 9.85 ± 5.73 in the young), they were not statistically significant. Similarly, Kuo et al. reported that the lumbar lordosis angle was 15.2 ± 9.3 in the elderly and 16.0 ± 5.6 in the young that was also not statistically significant [33]. In addition, our study was in line with Okpala et al. who found that the lumbar lordosis did not change significantly with aging in the normal population [4]. Furthermore, our correlation analysis did not show any significant correlation between age, BMI, and lumbar lordosis angle.

However, we found that the lumbar lordosis angle was significantly increased post the intervention. The magnitude of change in lumbar flexure in the young adults during standing was 35.13%; that in the older adults during standing it was 41.71%; and for the sitting position, the young adults showed 40.28% change in response to the intervention, which was higher than the effect reported in other studies [43, 44]. However, an exception was from Berjano et al. [45] that they reported a change in the lumbar lordosis angle of 121.82%.

The results of our study support the hypothesis that the lumbar lordosis curvature could be increased the cervical anterior inclination angle. This practice could be used as an alternative to improving the lumbar curvature by manipulating the pelvic forward inclination angle, especially under sitting conditions. Because the pelvis rotates backward when sitting, the psoas muscle tension decreases [46], and the lumbar lordosis decreases [47]. Schmidt and colleagues tried to improve lumbar curvature and relieve low back pain by correcting pelvic inclination, but the changes were not statistically significant [48]. In addition, whether in school or at home, school-age children spend more and more time in a sitting posture, and maintaining a sitting posture for a long time will lead to a decrease in lumbar lordosis and thoracic kyphosis [49]. The findings from our study also support this view, as the lumbar lordosis angle was significantly reduced in the sitting position compared with the standing position.

### Anteversion angle of the pelvis

Our results showed that understanding posture, the pelvic forward inclination was significantly different between the two age groups, where the young group (22.97 ± 5.01) was significantly higher than that of the older group (9.8 ± 3.22). Furthermore, the pelvic forward inclination angle was significantly and negatively correlated with age and BMI and positively correlated with height. Hu et al. studied asymptomatic Chinese adults and found that the male’s pelvic inclination was greater than the female in all age groups, and the pelvic inclination of older adults was greater than the young (young 11.5 ± 7.8, older 14.5 ± 9.5) [50]. Another study reported that, in the standing position, the pelvic retroversion angle of the elderly volunteers was larger than that of the young people, and the pelvic retroversion occurred from standing to sitting position [51]. These differences may be linked to age-related spinal degeneration because the thoracic kyphosis angle increases with age and is accompanied by a decrease in pelvic forward inclination to rebalance the spine.

There was a significant difference in pelvic forward inclination pre and post the intervention in standing posture. Some studies have shown that the increase in the anterior inclination of the pelvis is related to the decrease in the cervical angle [16], which is in line with our results, but because the locations of the markers for the cervical angle in this study were different from that study, when the cervical anterior inclination increased, the anteversion of the pelvis would also increase significantly. It has been reported that the increase in pelvic forward inclination in the standing position may also be related to an increase in lumbar extension [37]. However, some researchers hold the opposite view as they found that the increase in pelvic forward inclination was related to the degree of lumbar lordosis when sitting, but had no relation to the degree of lumbar lordosis when standing [16]. Unfortunately, because of instrument limitations, in this study we could not measure the pelvic inclination in the sitting position, which may be examined in future research.

### Limitations and future research direction

Results of the current study can only apply to healthy people and should not be generalized to populations with back pain and other neuromuscular disorders. In addition, the sample size is relatively small and we could not examine the differences in gender.

Although several studies have already explored the relationship between spine angles using external markers, there are significant discrepancies in the placement of the markers. To optimize the study on different spine angles and to facilitate comparison of the results between different studies, a more standardized method for spine angle analysis is needed.

Despite these limitations, as far as we know, this was still the first study aiming to improve the lumbar lordosis angle by adjusting the position of the head, which can provide a reference for the prevention and treatment of low back pain in the elderly and sedentary groups such as students.

## Conclusion

The results of this study indicated that increasing the cervical anterior inclination angle can increase the lumbar lordosis angle and reduce the thoracic kyphosis angle. Therefore, cervical anterior inclination can be used as an alternative to pelvic forward inclination to improve the lumbar lordosis angle.

## Supplementary Information

**Additional file 1.** Posture and muscle tension.

## Data Availability

The data in this study are available from the corresponding author upon request, if legally and ethically possible. Each author warrants that this work is original. Neither this work nor a similar work by the authors has been published elsewhere in any language nor shall be submitted for publication elsewhere while under consideration by European Spine Journal.

## Abbreviations

LL: Lumbar lordosis
LBP: Low back pain
CAA: Cervical anterior angle
LLA: Lumbar lordosis angle
TKA: Thoracic kyphosis angle
PFIA: Pelvic forward inclination angle
ASIS: Anterior superior iliac spine
PSIS: Posterior superior iliac spine
