# Supplementary material for: Effects of a postural cueing for head and neck posture on lumbar lordosis angles in healthy young and older adults: a preliminary study
Source: J Orthop Surg Res. 2022 Apr 4;17:199. doi: 10.1186/s13018-022-03090-9 (PMC8981642; doi:10.1186/s13018-022-03090-9)
Supplement: Supplementary file 1 — Additional file 1. Posture and muscle tension. [file 13018_2022_3090_MOESM1_ESM.pdf]

9784789920919
1920075068004
ISBN978-4-7899-2091-9
C0075 ¥6800E
定価：本体6,800円＋税

CONTENTS

1. Functional Neuro-muscular Conditioning
機能的神経—筋コンディショニング
2. Functional Joint Conditioning
機能的関節コンディショニング
3. Functional Mental Conditioning
機能的メンタルコンディショニング
4. Functional Recovery Conditioning
機能的リカバリーコンディショニング

現代では、トップアスリートから一般・高齢者にいたるまで、身体に何らかの「筋弱化」（抑制）の問題を抱えている方がほとんどです。これによって、気づかないうちに効率的な動きが妨げられ、運動のパフォーマンスは低下しています。《ホリスティック コンディショニング》では、「神経—筋機能」「関節機能」を整えて、全身の筋運動・連鎖および関節機能連鎖を最適な状態にし、さらにこれらの機能を最大限に発揮させるために「メンタル」「リカバリー（傷害防止）（回復促進）（栄養補給）」を適用しています。

ホリスティック コンディショニング 1
矢野雅知 著
ホリスティック＝〈総合的・包括的〉アプローチ
スキージャーナル

HOLISTIC CONDITIONING

ホリスティック コンディショニング

ホリスティック＝〈総合的・包括的〉アプローチ

NO.1

《ホリスティック コンディショニング》では、身体運動の本質的な動きを鍛えるFNC［機能的神経—筋コンディショニング］理論、関節を正しく機能させるFJC［機能的関節コンディショニング］理論をベースとし、問題を引き起こしている真の原因を正しく把握します。そして、一般・高齢者の運動効果を格段に高め、アスリートのコンディションをベストな状態へ導くために、さまざまな手技・テクニックを用いてアプローチしていきます。本書は、あらゆる運動指導の現場、スポーツの現場で欠かせない一冊となるはずです。

SKI journal

3 筋のアンバランスと身体の歪み

①肩の左右傾斜に関わる筋

《広背筋》イラスト30

左広背筋弱化

⇒左肩上がる

⇒頭部は水平

＊通常、弱化側の上部僧帽筋が緊張して、頭部も傾斜する。

《上部僧帽筋》イラスト31

左上部僧帽筋の弱化

⇒左肩下がる

⇒頭部反対側に傾斜

＊通常、対側の上部僧帽筋がショートとなる。

《下部僧帽筋》イラスト32

右下部僧帽筋の弱化

⇒右肩甲骨上がる

⇒右肩が前に回転

《菱形筋》イラスト33

左菱形筋の弱化

⇒左肩甲骨（肩）下がる

⇒頭部が左側に回転

《仙棘筋（脊柱起立筋群）》イラスト34

右仙棘筋の弱化

⇒右側逆Cカーブ

⇒右肩上がる

⇒右骨盤下がる

＊腹臥位では、右側が低くなる。

《中臀筋》イラスト35

右中臀筋の弱化

⇒右側の骨盤・肩・頭部上がる

イラスト30

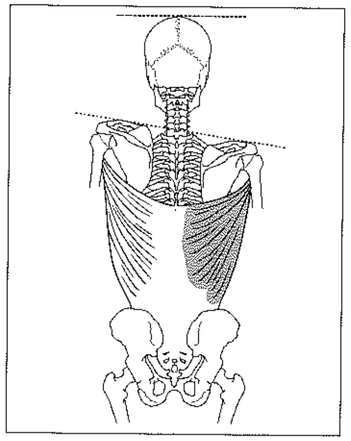

イラスト31

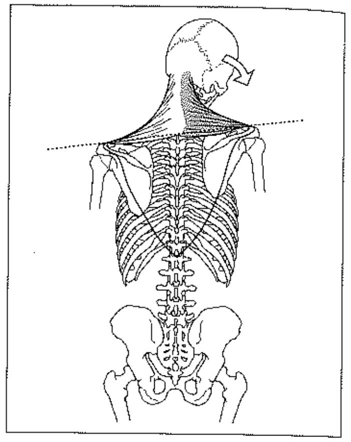

イラスト32

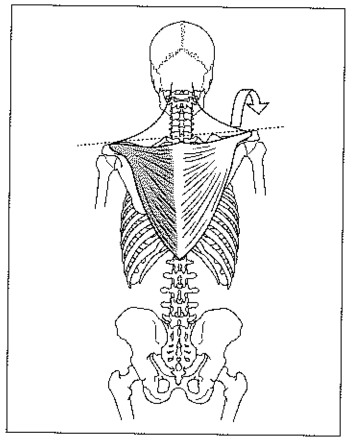

イラスト33

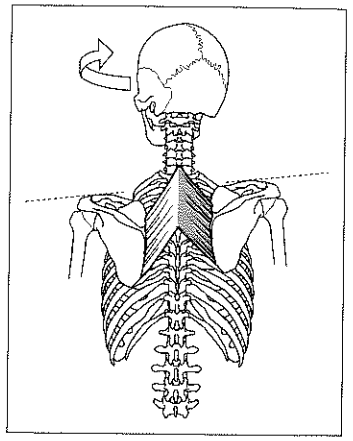

イラスト34

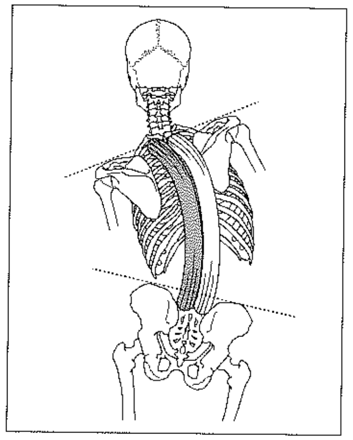

イラスト35

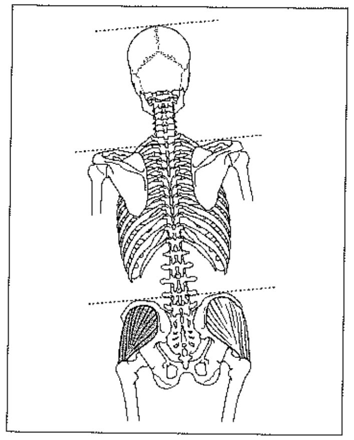

②骨盤の左右傾斜に関わる筋

《大臀筋》イラスト36

右大臀筋の弱化

⇒右骨盤上がる

⇒右骨盤前方回旋

⇒足・脚は内旋

《大腿筋膜張筋》イラスト37

右大腿筋膜張筋の弱化

⇒右骨盤上がる

＊右膝関節屈曲位⇒外側の固定力弱化

右膝関節伸展位⇒内反膝

《内転筋群》イラスト38

右内転筋群の弱化

⇒対側骨盤（左側）上がる

⇒弱化側（右）に内反膝

《腸腰筋（大腰筋・腸骨筋）》イラスト39

左腸腰筋の弱化

⇒弱化側（左）骨盤上がる

⇒緊張側（右）に腰椎の側屈・回旋

（⇒腰椎の側湾）

イラスト36

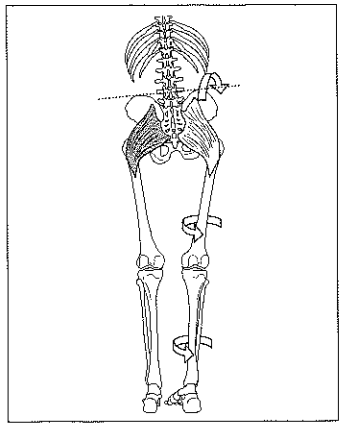

イラスト37

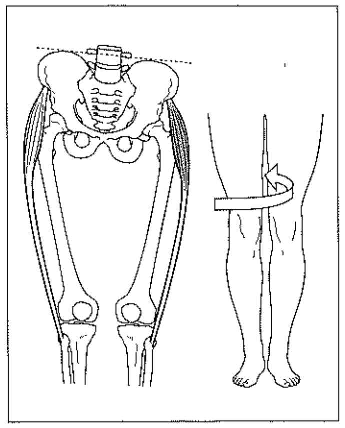

イラスト38

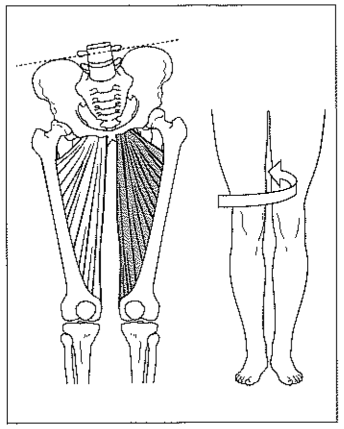

イラスト39

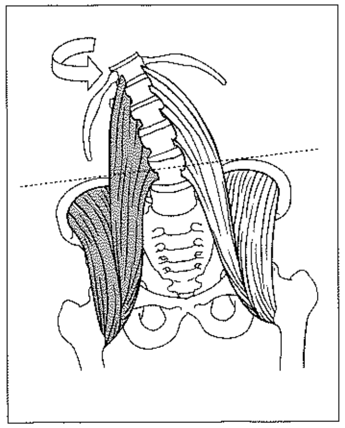

③腰椎側湾に関係する筋

《大腰筋》イラスト40

⇒片側の弱化で、腰椎が側湾する。

⇒両側弱化で、腰椎カーブの減少

（⇒骨盤後傾）

⇒両側過緊張（拘縮）で、腰椎過剰前湾

（⇒骨盤前傾）

《腰方形筋》イラスト41

右腰方形筋の弱化

⇒対側（左）の腰方形筋過緊張で、

腰椎の側屈・回旋

⇒弱化筋（右）の第12肋骨上がる

（⇒骨盤は水平）

イラスト40

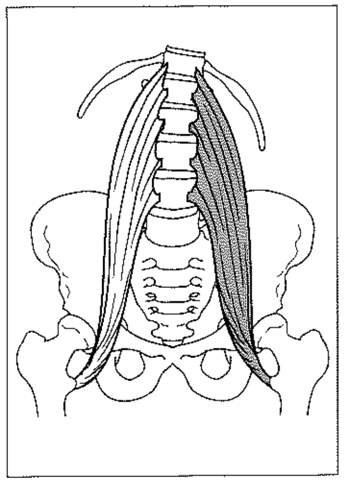

イラスト41

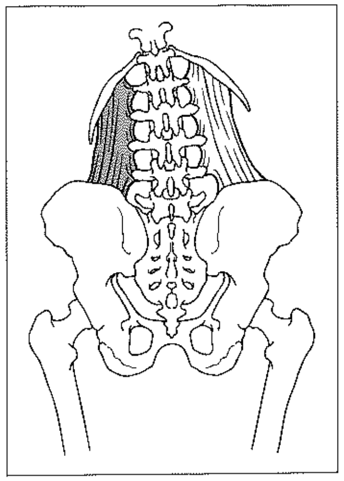

④骨盤・股関節に関係する筋

《ハムストリングス》イラスト42 ①

ハムストリングスが弱化すると⇒骨盤前傾

腹筋が弱化すると⇒骨盤前傾

《大腿直筋（大腿四頭筋）》イラスト42 ②

大腿直筋が弱化すると⇒骨盤後傾

《ハムストリングス》イラスト43

右:内側ハムストリングスが弱化すると⇒下腿の外旋

左:外側ハムストリングスが弱化すると⇒下腿の内旋

《縫工筋および薄筋》イラスト44

弱化側の腸骨のサポートが弱まり

⇒腸骨の後方回旋

⇒膝の不安定

《大臀筋》イラスト45

弱化すると

⇒腸骨の前方回旋

⇒膝関節外側の不安定

《梨状筋》イラスト46

弱化すると

⇒弱化側の大腿骨が内旋する

⇒弱化側の対側梨状筋が緊張して、仙骨を引っ張る

イラスト42

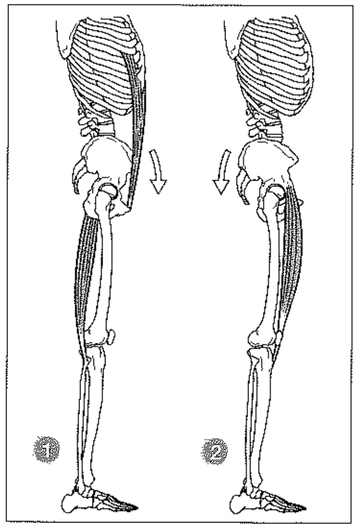

イラスト43

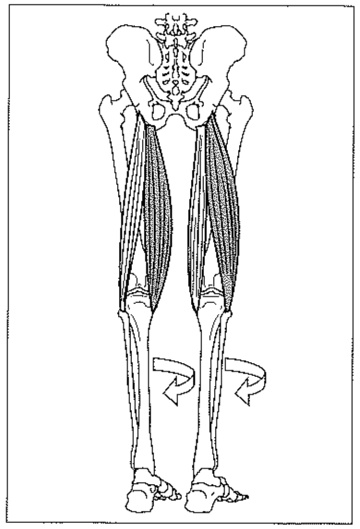

イラスト44

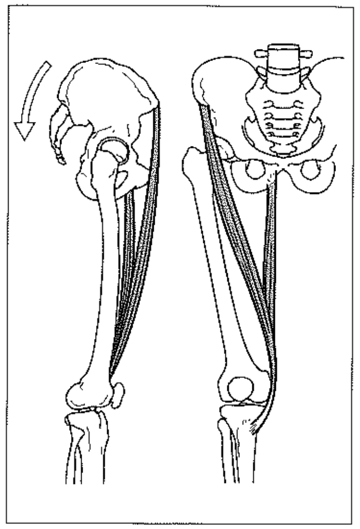

イラスト45

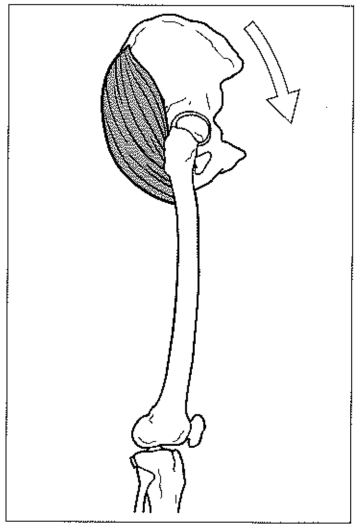

イラスト46

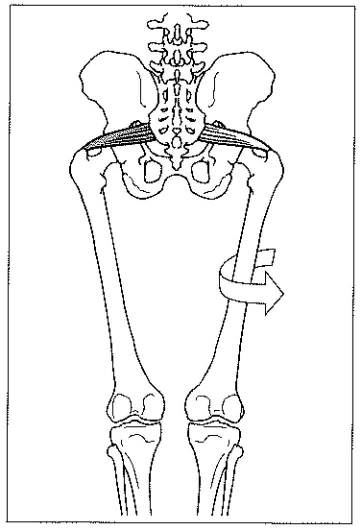

⑤胸椎・頚椎の前方傾斜に関わる筋

《中・下部僧帽筋》イラスト47

弱化すると

⇒胸椎サポート減少して、猫背となる

《頚椎伸筋群》イラスト47

弱化すると

⇒頭部の前方傾斜

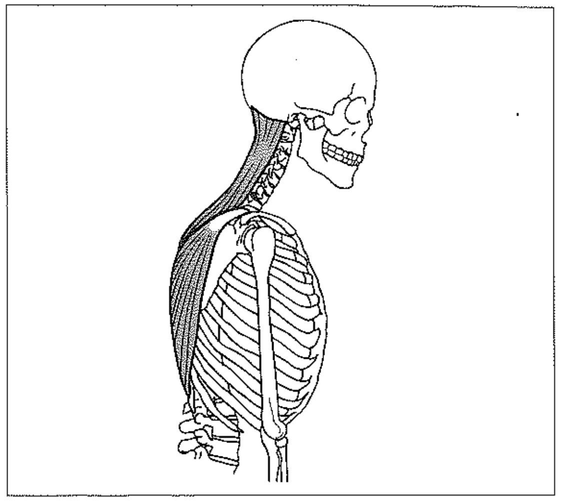

イラスト47

⑥翼状肩甲骨に関わる筋

《前鋸筋》イラスト48

菱形筋と前鋸筋の筋アンバランス

⇒前鋸筋弱化すると、プッシュアップで、肩甲骨が翼状に突出する

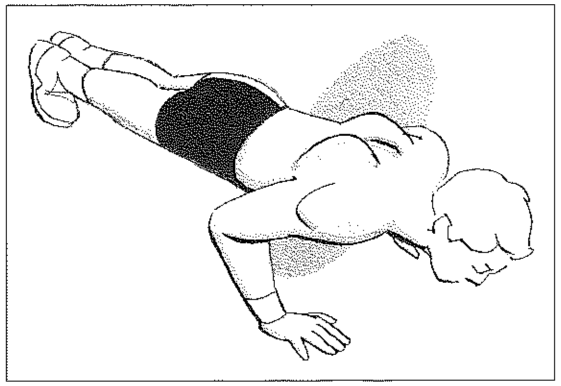

イラスト48

⑦頭部の回旋に関わる筋

《胸鎖乳突筋》イラスト49

胸鎖乳突筋が弱化すると

⇒弱化側に頭部が回旋する

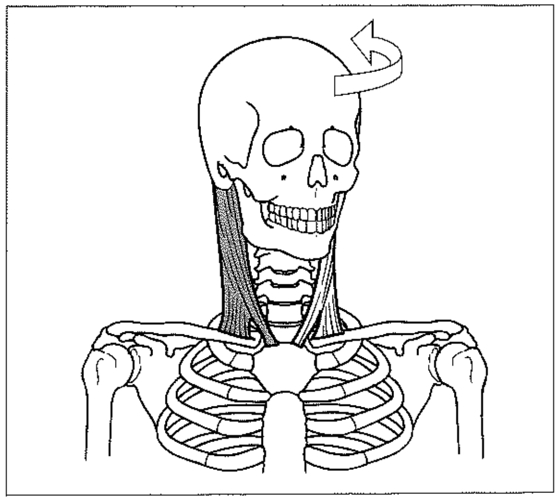

イラスト49

⑧腕の回外・回内に関する筋

《腕の回外（手の平前方傾向）》 イラスト50

弱化筋⇒三角筋鎖骨部（前部線維）大胸筋

過緊張⇒広背筋　大円筋

《腕の回内（手の平後方傾向）》 イラスト51

弱化筋⇒三角筋肩甲棘部（後部線維）　広背筋

大円筋　棘上筋・棘下筋・小円筋

過緊張筋⇒三角筋鎖骨部（前部線維）　大胸筋

肩甲下骨

イラスト50

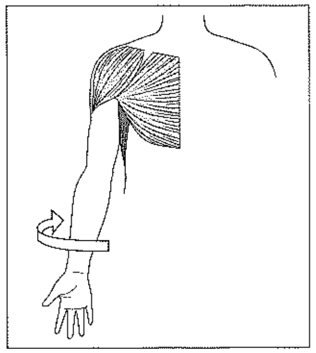

イラスト51

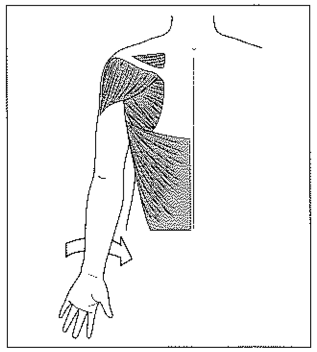

⑨O脚・X脚に関わる筋

《内反膝（O脚傾向）》 イラスト52

過緊張筋⇒内側ハムストリングス　中臀筋

弱化筋⇒外側ハムストリングス　大腿筋膜張筋

内転筋群　大臀筋

《外反膝（X脚傾向）》 イラスト53

過緊張筋⇒大腿筋膜張筋　内転筋群　大臀筋

外側ハムストリングス

弱化筋⇒内側ハムストリングス　中臀筋

*オーバートレーニング症候群の兆候を示し、副腎機能低下（胸椎9番変位）が示されるケースでは、縫工筋・薄筋の弱化側の骨盤前方のサポートが失われて、膝が不安定となる。

イラスト52

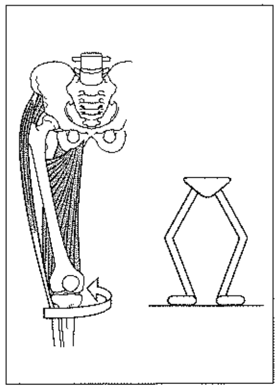

イラスト53

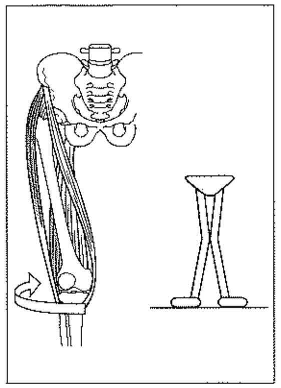

⑩下腿・足部に関わる筋

《ヒラメ筋》 イラスト54

弱化すると⇒身体の前方傾斜

《腓腹筋》 イラスト55

弱化すると⇒反張膝（膝の過伸展）　身体の前方傾斜

《長・短腓骨筋（右足）》 イラスト56

右足が弱化すると⇒足が回外（内反）する

《後脛骨筋・前脛骨筋（左足）》 イラスト56

左足が弱化すると⇒足が回内（外反）する

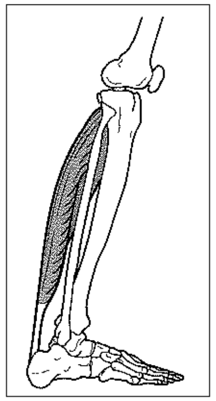

イラスト54

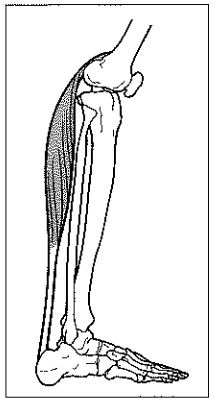

イラスト55

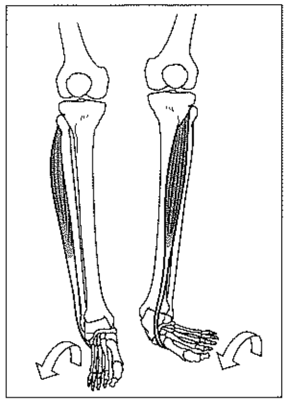

イラスト56

筋のアンバランスによる姿勢変位

表9 に、姿勢変位からみた筋の弱化をまとめた。

表9 筋のアンバランスによる姿勢変位

部位	関係する弱い筋	備考
頭部の左右傾斜	頸椎筋、菱形筋、仙棘筋、僧帽筋上部、広背筋、（腸腰筋、小・中臀筋）	菱形筋：弱い側の肩甲骨下がり、弱い側に頭部回転<br>僧帽筋上部：弱い側の肩が下がる
肩の左右傾斜	広背筋、僧帽筋上部、頸椎筋（三角筋）（小・中臀筋、腸腰筋）	
肩の前後回転	肩甲下筋、広背筋、僧帽筋下部、菱形筋、腹斜筋	僧帽筋下部：弱い側の肩甲骨上がる
肩の片方下がり	僧帽筋上部・中部・下部、菱形筋、肩甲挙筋	
腕の回内（外旋筋弱）	後部三角筋、棘上筋、棘下筋、小円筋	掌後向き
腕の回外（内旋筋弱）	前部三角筋、大胸筋、広背筋、大円筋、肩甲下筋	掌前向き
肩甲骨	前鋸筋：弱いとウイング外に出る<br>僧帽筋上部：弱い側に下がる／下部：弱い側が上がる	
腰の左右傾斜	腸腰筋、小・中臀筋、腰方形筋、大臀筋、大腿筋膜張筋、仙棘筋、腹筋	大腿筋膜張筋：弱い側の骨盤上がり、内反膝となる
骨盤の前方傾斜	大臀筋、ハムストリングス、大腿筋膜張筋、下部仙棘筋、腹筋	過緊張の腸腰筋<br>内転筋：弱い側は内反膝となり、反対側の骨盤が上がる
骨盤の後方傾斜	縫工筋、腸腰筋、大腿筋膜張筋、大腿直筋、下部仙棘筋	縫工筋・薄筋：弱いと骨盤を前方にサポートできない（腸骨後方変位）
側湾Cカーブ	腹筋、仙棘筋、広背筋、腹斜筋	
脊椎　頸椎<br>胸椎<br>腰椎	頸椎伸展筋：弱いと頭部の前方傾斜<br>僧帽筋下部：（猫背）胸椎後湾<br>大臀筋：弱いと腰椎前湾・膝の不安定	左右の腸腰筋：弱いと腰椎カーブ減少<br>ハムストリングス：弱いと腰椎前湾（坐骨後方変位）<br>腹筋弱いと腰椎前湾
外反膝（X脚）	縫工筋、薄筋、中臀筋、大臀筋、内転筋	過緊張の大腿筋膜張筋<br>縫工筋・薄筋は骨盤のバランス
内反膝（O脚）	大腿筋膜張筋、大臀筋、内転筋	内転筋：弱い側は内反膝となり、反対側の骨盤が上がる
膝の過伸展（反張膝）	膝下筋、腓腹筋、内側・外側ハムストリングス（大腿二頭筋）	外側ハムストリングス：弱いと内反（O脚）／内側ハムストリングス：弱いと外反（X脚）<br>片脚のハムストリングス：弱いと骨盤の回転
内がえし	腸腰筋、長・短腓骨筋、梨状筋、内転筋、外側ハムストリングス（大腿二頭筋）	
外がえし	内側ハムストリングス、薄筋	過緊張の梨状筋
外反足<br>ハイアーチ	長・短腓骨筋	長・短腓骨筋：弱いとくるぶし・足の内旋
内反足<br>偏平足	前脛骨筋、梨状筋、外側ハム（大腿二頭筋）腸腰筋	前脛骨筋弱いと足の外旋
身体全体の前方傾斜	ヒラメ筋	ヒラメ筋：弱いと足首が背屈<br>腓腹筋：弱いと足首が底屈（反張膝）<br>＊ヒラメ筋・腓腹筋：硬いと足首制限受けて歩行時に踵から足先に体重を移すときにトゥー・アウトする
